# Current State of Evidence for Use of MRI in LI‐RADS

**DOI:** 10.1002/jmri.29748

**Published:** 2025-02-21

**Authors:** Ameya Madhav Kulkarni, Danielle Kruse, Kelly Harper, Eric Lam, Hoda Osman, Danyaal H. Ansari, Umaseh Sivanesan, Mustafa R. Bashir, Andreu F. Costa, Matthew McInnes, Christian B. van der Pol

**Affiliations:** ^1^ Department of Medical Imaging, Hamilton Health Sciences McMaster University Hamilton Ontario Canada; ^2^ Department of Diagnostic Imaging Juravinski Hospital and Cancer Centre, Hamilton Health Sciences Hamilton Ontario Canada; ^3^ Departments of Radiology and Medicine Duke University Medical Center Durham North Carolina USA; ^4^ Department of Radiology The Ottawa Hospital, University of Ottawa Ottawa Ontario Canada; ^5^ Ottawa Hospital Research Institute Clinical Epidemiology Program Ottawa Ontario Canada; ^6^ Department of Diagnostic Radiology, Kingston Health Sciences Centre Kingston General Hospital Kingston Ontario Canada; ^7^ Center for Advanced Magnetic Resonance Development Duke University Medical Center Durham North Carolina USA; ^8^ Queen Elizabeth II Health Sciences Centre and Dalhousie University Halifax Nova Scotia Canada

**Keywords:** carcinoma, diagnostic techniques, digestive system, hepatocellular, liver, magnetic resonance imaging, observer variation

## Abstract

The American College of Radiology Liver Imaging Reporting and Data System (LI‐RADS) is the preeminent framework for classification and risk stratification of liver observations on imaging in patients at high risk for hepatocellular carcinoma. In this review, the pathogenesis of hepatocellular carcinoma and the use of MRI in LI‐RADS is discussed, including specifically the LI‐RADS diagnostic algorithm, its components, and its reproducibility with reference to the latest supporting evidence. The LI‐RADS treatment response algorithms are reviewed, including the more recent radiation treatment response algorithm. The application of artificial intelligence, points of controversy, LI‐RADS relative to other liver imaging systems, and possible future directions are explored. After reading this article, the reader will have an understanding of the foundation and application of LI‐RADS as well as possible future directions.

## 1 Background

The American College of Radiology (ACR) Liver Imaging Reporting and Data System (LI‐RADS) is a standardized system for liver imaging in patients at high risk for hepatocellular carcinoma (HCC) that is supported and endorsed by the American Association for the Study of Liver Diseases (AASLD), the Organ Procurement and Transplantation Network (OPTN), and is consistent with the National Comprehensive Cancer Network (NCCN) guidelines for HCC [1]. LI‐RADS includes standardized terminology, technique, interpretation, reporting, and data collection to help improve communication, patient care, education, and research. The initial version of LI‐RADS was introduced in 2011 after a small group of imaging experts convened to address the need for improved consistency and clarity of liver imaging reporting, modeled on the Breast Imaging Reporting and Data System (BI‐RADS) but for categorization of the probability of HCC [2, 3]. Several updates have since been released, driven by accumulated data and user feedback, including v2013.1, v2014, Contrast‐Enhanced Ultrasound (CEUS) v2016, CT/MRI v2017, CEUS v2017 Essentials, and Ultrasound (US) core 2017 [1]. There are currently six LI‐RADS algorithms including for surveillance (US Surveillance v2024 Core), diagnosis (CT/MRI v2018 and CEUS v2017) and assessing response to treatment (CT/MRI Nonradiation TRA v2024, CT/MRI Radiation TRA v2024, CEUS Nonradiation TRA v2024).

LI‐RADS is applicable to a large subset of patients at high risk of developing HCC and includes patients with cirrhosis from non‐vascular etiologies, chronic hepatitis B viral (HBV) infection, and current or prior HCC [4]. It is important to limit the application of LI‐RADS to this population as the vast majority of LI‐RADS evidence is based on these patients. Limiting LI‐RADS to these patients is necessary for LI‐RADS 5 (LR‐5) to maintain a high specificity for HCC, for which biopsy is not necessary, and instead the patient can proceed directly to treatment.

In this article, we review the pathogenesis of HCC, the use of MRI in LI‐RADS, and provide a detailed review of the LI‐RADS diagnostic algorithm. Its main components, including major and ancillary features, as well as the LR‐M (malignancy) and LR‐TIV (tumor in vein) categories, are reviewed. The reproducibility of the diagnostic algorithm is discussed, followed by a review of the treatment response algorithm, the application of artificial intelligence (AI) and radiomics to LI‐RADS, points of controversy, LI‐RADS relative to other liver imaging systems, and possible future directions for LI‐RADS.

## 2 HCC Pathogenesis

Primary risk factors for HCC include liver cirrhosis and chronic hepatitis B; however, nonviral risk factors are becoming more common, including metabolic‐dysfunction‐associated steatotic liver disease [5]. Chronic hepatic inflammation eventually results in progressive epigenetic and genetic alterations [6, 7]. Mutations result in the clonal propagation of molecular abnormalities [8]. Hepatocarcinogenesis refers to a progressive cellular and molecular dedifferentiation of hepatocytes that occurs via complex processes that result in the formation of neoplastic hepatocytes [9]. HCC can develop de novo or in a multistep pathway. De novo HCC emerges without histologically definable precursor lesions. Multistep HCC goes through a series of transformations with varying degrees of aggressiveness. One or more steps of hepatocarcinogenesis may be skipped. When multiple HCCs develop in a liver simultaneously, it is referred to as multicentric HCC.

One‐third of HCC can be classified by its molecular subtype into one of eight categories: steatohepatitic, clear cell, macrotrabecular‐massive, scirrhous, chromophobe, fibrolamellar, neutrophil‐rich, and lymphocyte‐rich, each of which can demonstrate specific imaging features described elsewhere [10, 11]. The remaining approximately two‐thirds of HCCs demonstrate molecular features that are not otherwise specified based on the WHO 2019 classification of tumors 5th edition [12].

Intrahepatic cholangiocarcinoma can arise from similar oncogenic conditions in patients with chronic liver disease and account for approximately 15% of all primary liver cancers [13]. Some tumors exhibit features of both HCC and intrahepatic cholangiocarcinoma [14].

## 3 MRI in LI‐RADS

Dynamic contrast‐enhanced CT and MRI are commonly used for the assessment of HCC, with growing use of CEUS. Of these, MRI has the advantages of superior soft tissue contrast and depiction of more ancillary features of HCC compared to the other two modalities [15]. Both CT and MRI provide volumetric coverage of the liver during dynamic phases, while CEUS sacrifices coverage for much higher temporal resolution.

Along with the ability to depict additional static features not available via CT (e.g., intralesional iron, T_2_ hyperintensity, and restricted diffusion), MRI can provide hepatobiliary‐phase imaging using specialized contrast agents, further characterizing liver observations. Ultimately, MRI has been shown to have equivalent to superior diagnostic accuracy when directly compared with CT, particularly for small liver observations [16, 17, 18].

However, the advantages of MRI are not without drawbacks. MRI examinations are more expensive than either CT or CEUS, have unique facility requirements, and involve other factors. In particular, scan time is becoming a greater drawback in the modern era of burgeoning patient populations and limited medical resources. Furthermore, leveraging MRI's ability to depict liver observations using many different sequences leads to more images and longer acquisition and interpretation times. Strategies have been advanced to perform abbreviated MRI either for HCC surveillance or combined surveillance and diagnosis, but these involve limiting certain portions of the MRI examination, and their role in the care of patients at risk for HCC is not yet clearly defined [19, 20, 21].

Finally, the use of hepatobiliary contrast agents remains an area of controversy and deserves special consideration. Hepatobiliary phase imaging has been shown to improve both the detection of small focal observations and the characterization of observations, particularly benign observations such as transient perfusional anomalies and shunts [22]. However, the use of these agents also involves substantial trade‐offs. Hepatobiliary agents are taken up relatively quickly by the liver but can still require 15–20 min to achieve an adequate hepatobiliary phase; thus, the final post‐contrast images are obtained 15–20 min following contrast injection, compared with 3–5 min for extracellular agents. Additionally, hepatobiliary uptake of contrast begins within a few minutes of administration, creating overlap with the delayed vascular phase and confounding the interpretation of post‐contrast phases between approximately 3–10 min. Finally, these agents can increase the incidence of artifacts during the critical arterial phase acquisition, namely respiratory motion artifact, and while these can be mitigated using advanced imaging techniques such as single‐breath‐hold multiple arterial phase acquisitions, they remain an issue when advanced techniques are not available [23]. As a result, there remains disagreement regarding the ideal choice of contrast agent for the surveillance and diagnosis of HCC at MRI.

## 4 CT/MRI LI‐RADS Diagnostic Algorithm

The CT/MRI LI‐RADS diagnostic algorithm was created for a high level of specificity and positive predictive value for HCC, negating the need for biopsy for LR‐5 lesions. Each liver observation is categorized separately, rather than assigning one category for the liver as a whole [24]. The categories have been found to carry the following positive predictive values (PPV) for HCC on MRI using extracellular and hepatobiliary contrast agents as follows, respectively: LR‐1: benign, 0%; LR‐2: probably benign, 6% and 1%; LR‐3: intermediate, 31% and 38%; LR‐4: probably HCC, 64% and 77%; LR‐5: definitely HCC, 95% and 96% [25]. LR‐M, which is discussed separately, includes both HCC and non‐HCC malignancies, as does LR‐TIV [1].

To apply the algorithm for MRI, the patient must first be categorized as high‐risk for HCC meeting LI‐RADS inclusion criteria, and an appropriate MRI protocol then needs to be obtained. If a liver observation (or observations) is identified, the algorithm is applied, starting with the exam quality and excluding liver observations that are incompletely evaluated such as from suboptimal arterial phase timing (LR‐NC). Additionally, observations that have been treated previously are assessed using the Treatment Response Algorithm (TRA), discussed below. While some guidance is provided for identifying benign features, this step is performed mainly based on pattern matching and gestalt, rather than structured application of the algorithm (LR‐1 and LR‐2). Characterization of common benign entities as LR‐1 (definitely benign) using their characteristic imaging features is performed for findings such as cysts, focal fat, and hemangiomas. Vascular pseudolesions, arterioportal shunts, or perfusional alterations demonstrate arterial enhancement but have no correlate on all other sequences. These are often wedge‐shaped and located at the liver periphery, without mass effect, and should be categorized as LR‐1 or LR‐2 (probably benign) [1]. Malignant diagnoses that may include, but are not specific to, HCC are categorized as LR‐M, and tumor in vein is categorized as LR‐TIV, both discussed later in more detail. Finally, if a liver observation is not included in one of the above categories, major and optional ancillary features are applied to arrive at LR‐3 through LR‐5.

**FIGURE 1:**
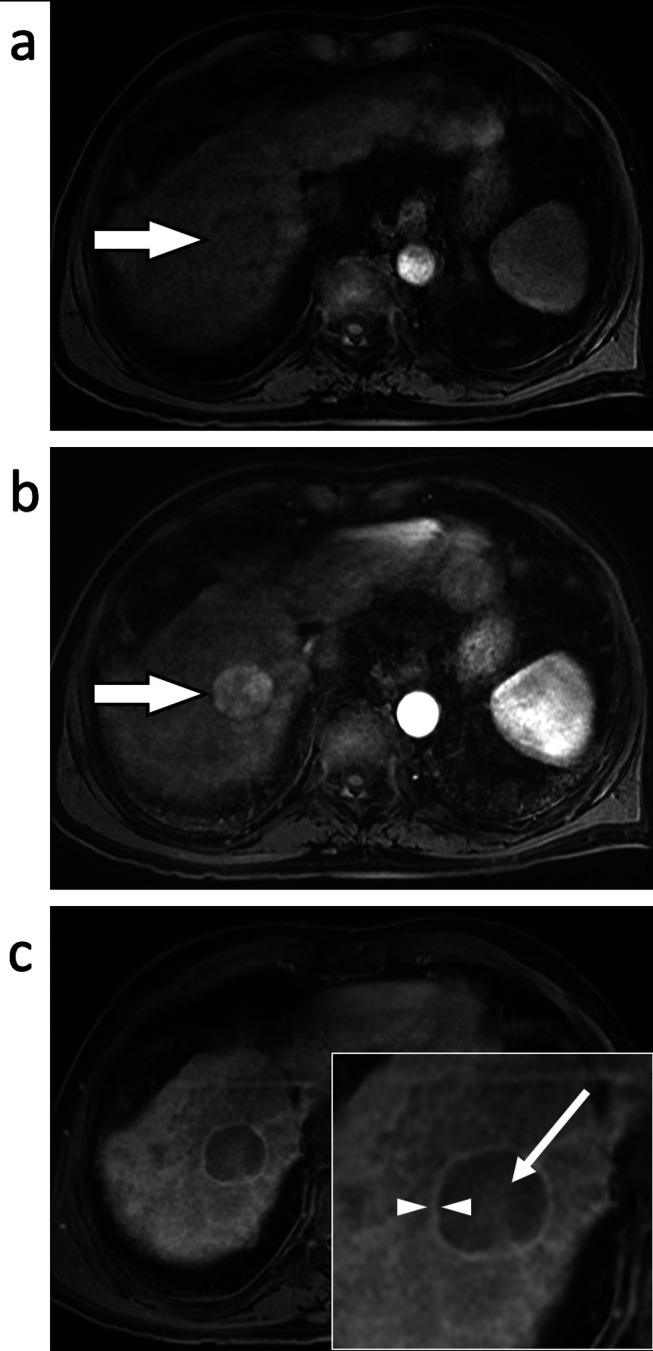
Axial T_1_‐weighted fat‐suppressed imaging of the liver in the unenhanced (a), arterial phase (b) and portal venous phase (c). These demonstrate a liver with a nodular contour from cirrhosis with a right hepatic lobe mass (a, arrow) that demonstrated arterial phase hyperenhancement (b, arrow) followed by portal venous phase (c) nonperipheral washout (arrow) and enhancing capsule (between arrowheads). This observation was categorized as LR‐5 (definitely HCC).

**FIGURE 2:**
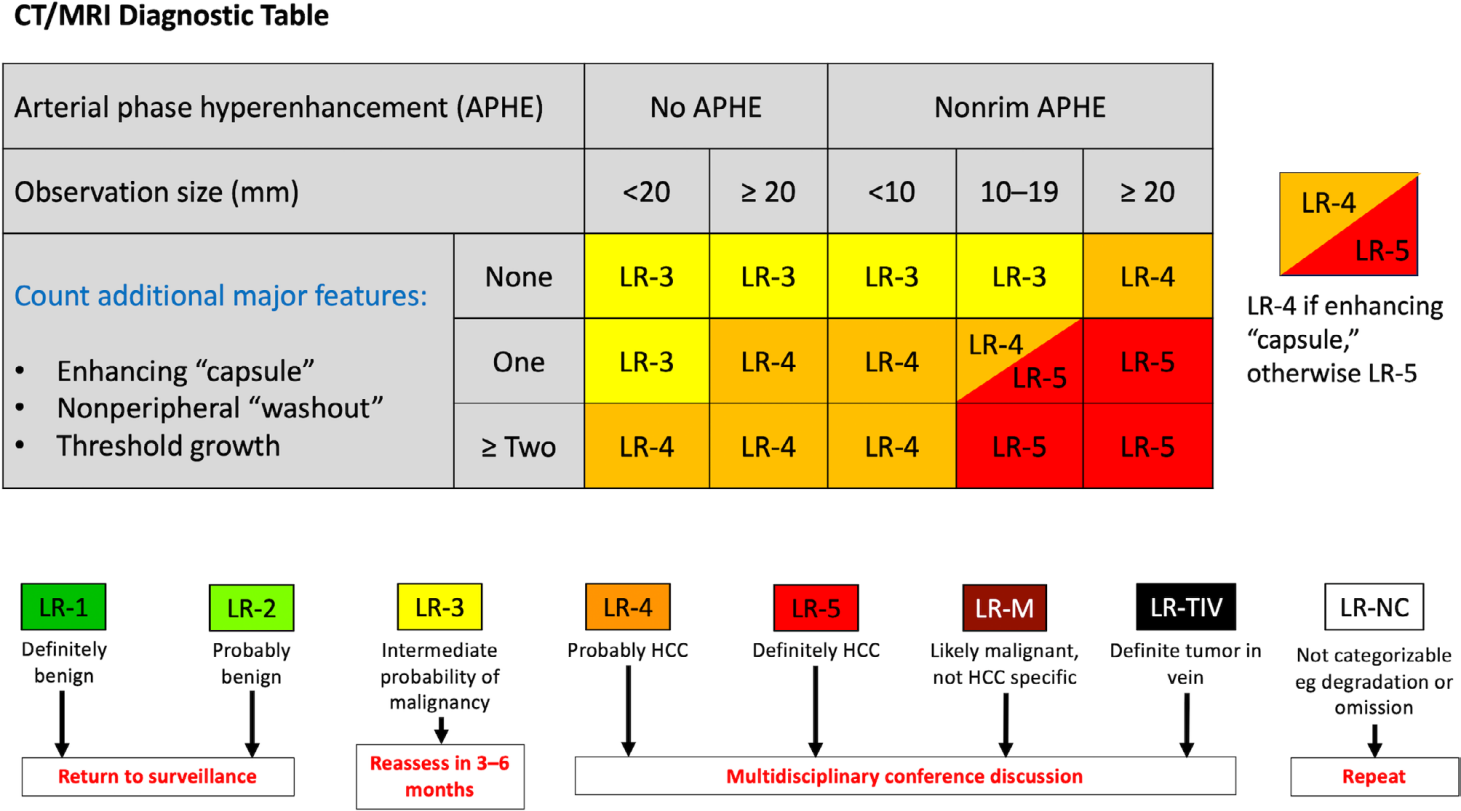
The American College of Radiology CT/MRI LI‐RADS version 2018 Diagnostic Table (Available at https://www.acr.org/Clinical‐Resources/Clinical‐Tools‐and‐Reference/Reporting‐and‐Data‐Systems/LI‐RADS). Below the table are descriptors of each category along with a summary of the corresponding management recommendation in red text.

The major features contributing to the LR‐3 through LR‐5 designations are: nonrim arterial phase hyperenhancement (nonrim APHE), nonperipheral washout, enhancing capsule (Figure 1), size (measured as the largest diameter), and threshold growth [1]. In the algorithm, starting with the presence or absence of nonrim APHE, then observation size, additional major features are counted to contribute to categorization (Figure 2). For example, an observation less than 20 mm with no APHE and no other features is an LR‐3. Only major features can contribute to an LR‐5 designation, and the observation must meet a minimum size of 10 mm and have nonrim APHE [26]. In general, if the presence of a major feature is uncertain, the feature should be assigned as “absent.”

**FIGURE 3:**
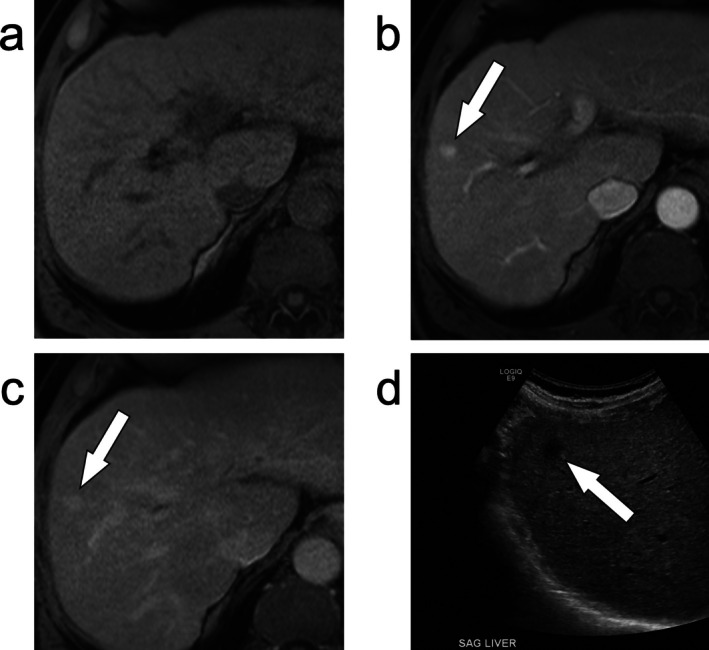
An axial T_1_‐weighted fat‐suppressed image of the liver (a) does not demonstrate any liver observations. Following the administration of intravenous gadolinium, a 1 cm focal observation demonstrated enhancement that persisted on the portal venous phase (b, arrow) and delayed phase (c, arrow). This fulfilled LR‐3 criteria (intermediate probability of malignancy) based on the major feature table. However, a prior ultrasound showed a corresponding lesion (d, arrow), which is an ancillary feature favoring malignancy in general, not HCC in particular. This observation was categorized as LR‐4 (probably HCC).

**FIGURE 4:**
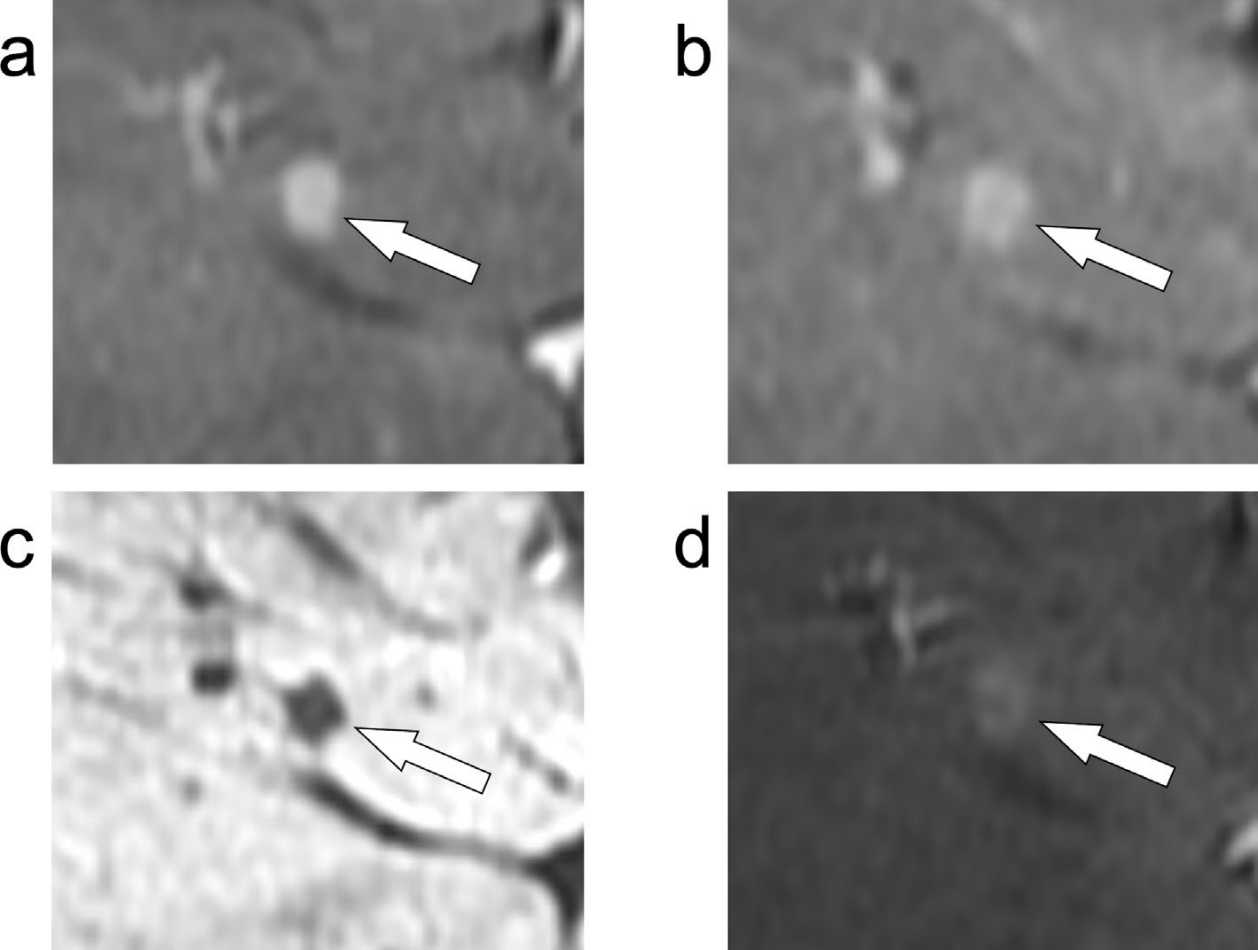
Axial T_1_‐weighted fat‐suppressed imaging of the liver demonstrates an observation with arterial phase hyperenhancement (a, arrow) without nonperipheral washout or an enhancing capsule on the portal venous phase (b, arrow) that fulfills LR‐3 criteria (intermediate probability of malignancy) based on the major features. The observation demonstrates hepatobiliary phase hypointensity (c, arrow) which is an ancillary feature favoring malignancy in general, not HCC in particular, which could be used to increase the category to LR‐4 (probably HCC). However, a prior MRI from over 3 years earlier demonstrated this observation to be stable in size (d, arrow). Size stability > 2 years is an ancillary feature favoring benignity that negates the ancillary feature favoring malignancy. The final categorization of this observation remained LR‐3.

Ancillary features (AFs) are optional and designed to improve accuracy while preserving HCC specificity. These features are separated into those favoring benign, malignant, and HCC‐specific categories. A feature favoring benignity may be used to downgrade a liver observation. To preserve the specificity of LR‐5 for HCC, an AF favoring HCC may not be used to upgrade from LR‐4 to LR‐5; however, a feature favoring benignity may be used to downgrade from LR‐5 to LR‐4. After these steps, if there is uncertainty regarding whether an observation belongs to one of two categories, the LI‐RADS tie‐breaking rules advise assignment to the category that implies greater uncertainty, and in the numerical categories, this typically means moving toward LR‐3 (Figures 3 and 4).

## 5 Major Features in the Diagnostic Algorithm

Each major feature in the LI‐RADS system—nonrim APHE, nonperipheral washout, enhancing capsule, size, and threshold growth—is applied to increase the accuracy of imaging‐based diagnosis of HCC. A 2018 systematic review by Tang et al. summarizes the evidence supporting these features, with nonrim APHE consistently noted as a highly sensitive finding of HCC, though its specificity can vary [27]. Washout appearance, when combined with APHE, enhances specificity but tends to decrease sensitivity. Enhancing capsule, while useful in some settings, has been debated for its inconsistent reporting and diagnostic utility across studies [27]. Threshold growth, a major LI‐RADS feature, is particularly challenging to assess due to its reliance on a prior imaging study for comparison. In an individual participant data (IPD) meta‐analysis from the LI‐RADS IPD Group, many original studies were found not to include prior imaging, and as a result, threshold growth reporting was only available for 754 of a total of 3414 CT observations (22%) and 1819 of a total of 11,532 MRI observations (16%) from the entire IPD database [28].

The LI‐RADS IPD Group has performed multiple IPD meta‐analyses, including an IPD meta‐analysis of 32 studies with 1170 liver observations on CT, 3341 on MRI, and 853 on CEUS, to establish the contributions of major features to the diagnostic algorithm by obtaining their independent associations with HCC [29]. The multivariable analysis found that all CT/MRI LI‐RADS major features except threshold growth (odds ratio [OR], 1.6; 95% confidence intervals [CI], 0.8–3.4; *p* = 0.19) were independently associated with HCC.

There is a need for a more consistent approach to reporting threshold growth, ensuring that its absence is accurately noted only when prior imaging is unavailable. Future studies might benefit from prioritizing the collection of longitudinal data that enables the assessment of threshold growth and explores how its combination with other LI‐RADS features may improve diagnostic performance. In general, data on the performance of combinations of LI‐RADS major features are lacking and mostly focus on the combination of nonrim APHE with washout [30, 31, 32].

## 6 Ancillary Features in the Diagnostic Algorithm

There are 21 CT/MRI AFs (version 2018) and five CEUS AFs (version 2017); these are divided into categories that favor malignancy in general, favor HCC in particular, or favor benignity. AFs may alter LI‐RADS categorization in 10%–35% of observations [33, 34]. Since their use is optional and inter‐reader agreement is poor to moderate [35, 36], AFs represent a source of inter‐reader variability. AFs also represent a potential source of inter‐modality variability, as some are unique to or more conspicuous on MRI, including three AFs exclusive to MRI with hepatobiliary contrast agent.

Evidence regarding the diagnostic performance of LI‐RADS AFs is scant compared with that for the major features. Studies have been limited by incomplete and heterogeneous datasets evaluating only subsets of AFs and small sample sizes, which is in part due to the rarity of some AFs [37]. Results from studies evaluating the impact of AFs on the diagnostic performance of CT/MRI LI‐RADS have been mixed. In a study that evaluated 102 patients with 275 liver observations using MRI with an extracellular contrast agent, Cerny et al. reported that AFs increased sensitivity while preserving high specificity for the diagnosis of HCC in categories LR‐4, LR‐5, and LR‐TIV [34]. However, the 95% CI of these estimates overlapped. In another study, the application of AFs increased the sensitivity of the LR‐4 category but decreased specificity [38]. Shropshire et al. evaluated 141 LR‐3 observations imaged with CT or MRI in 79 patients and found only 3/14 AFs were present in at least 5% of observations by consensus; none of the analyzed AFs predicted longitudinal changes in observation category [36]. In contrast, other studies have found improved diagnostic performance with some AFs, particularly diffusion restriction, mild–moderate T_2_ hyperintensity, and hypointensity in the transition and/or hepatobiliary phases [39, 40, 41, 42].

Recently, a large IPD meta‐analysis showed that almost all CT/MRI LI‐RADS AFs were independently associated with malignancy, HCC, and benignity, as intended by LI‐RADS [37]. However, this study did not evaluate the added value of AFs to the diagnostic performance of LI‐RADS. van der Pol et al. evaluated the impact of MRI‐based AFs on 222 observations in 81 patients [43]. Based on results from a machine learning model, the authors found that 13/21 AFs were noncontributory, and found no difference in area‐under‐the‐curve (AUC) between models with and without inclusion of AFs [43]. These findings suggest that, although AFs are associated with HCC and malignancy, there is redundancy between major and ancillary features, and their impact may be low.

Given that AFs add complexity, are optional to use, and may increase inter‐reader and inter‐modality variability, their utility to LI‐RADS is unclear. More evidence on the added diagnostic value of AFs is required. This requires larger studies with complete reporting of all major and ancillary features, which would enable the application of category adjustment rules and evaluation of overall LI‐RADS performance [28, 32].

## 7 LI‐RADS M and LI‐RADS TIV

LR‐M was created as a separate category in the diagnostic algorithm for liver observations that are likely to be malignant, but with imaging features not specific for HCC. Patients with cirrhosis or chronic hepatitis B are also predisposed to cholangiocarcinoma, albeit to a lesser extent, and cholangiocarcinoma does not exhibit the same imaging features as HCC [44]. In addition, some primary liver tumors represent bi‐phenotypic combined HCC –cholangiocarcinoma, exhibiting a combination of imaging features [14]. Because the LI‐RADS algorithm prioritizes specificity, observations that exhibit features suggestive of malignancy, but not necessarily HCC, are categorized as LR‐M, and typically undergo biopsy for definitive diagnosis [45]. The LR‐M category allows for high sensitivity in identifying malignancy (PPV: 93%–100% for malignancy, 30% and 35% for HCC using extracellular and hepatobiliary agent respectively), while preserving the specificity of LR‐5 for HCC [25].

**FIGURE 5:**
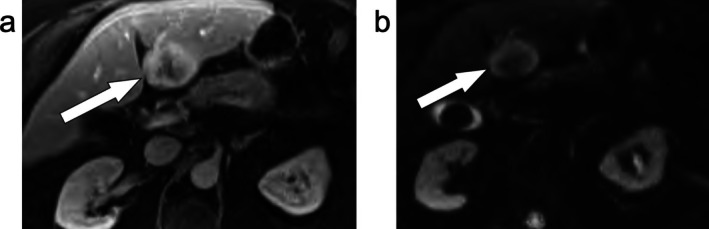
Axial T_1_‐weighted fat‐suppressed post‐contrast image of the liver (a) demonstrates a mass with rim enhancement (arrow). Axial diffusion weighted imaging (b) demonstrates this mass to have targetoid restriction (arrow). This mass was categorized as LR‐M and was confirmed as an intrahepatic cholangiocarcinoma.

**FIGURE 6:**
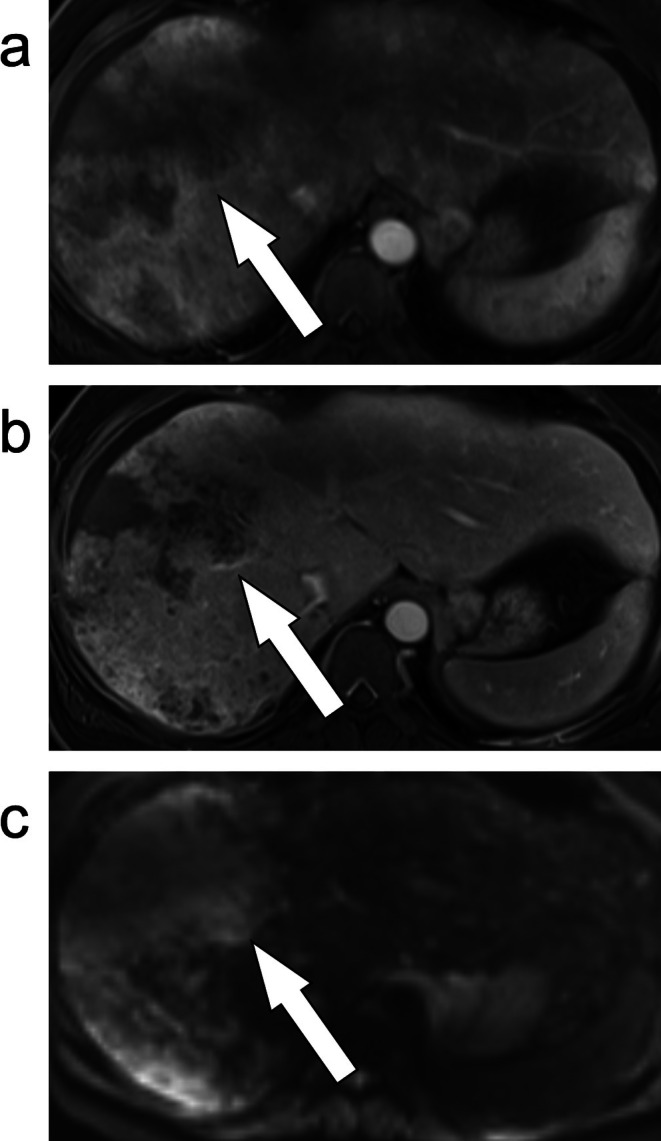
Axial arterial phase (a) and portal venous (b) phase post‐contrast T_1_‐weighted fat‐suppressed MR images, as well as diffusion weighted imaging (c) demonstrate an infiltrative appearance of an intrahepatic cholangiocarcinoma categorized as LR‐M (arrows). Infiltrative HCC can appear similarly.

The typical imaging features of mass‐forming intrahepatic cholangiocarcinoma on MRI have been adopted to define the LR‐M category, specifically the “targetoid features” of this malignancy (Figure 5). These features perform the best in identifying non‐HCC malignancy, particularly rim APHE, which has a 71% sensitivity and 85% specificity for non‐HCC malignancy [46]. Inter‐reader agreement is also moderate to substantial for these features, which is important given the fact that only a single feature is needed to assign the LR‐M category [47]. The nontargetoid features such as “infiltrative appearance” (Figure 6) included in LR‐M are slightly less accurate at identifying other malignancies compared to atypical HCC [46]. The algorithm recognizes this and suggests assigning LR‐M for infiltrative appearance, while stating “probably represents HCC.” [1] Overall, roughly one third of LR‐M lesions are atypical HCC, 1/3 are cholangiocarcinoma, and 1/3 are other lesion types [48].

The LR‐TIV category describes the presence of enhancing tumor in vein and can apply to observations that may represent HCC but may also be seen in other aggressive tumors, such as cholangiocarcinoma. Approximately 71% of lesions categorized as LR‐TIV are HCC [49], which explains the need for a separate category. Additionally, HCC with tumor in vein is associated with worse clinical outcomes and is a contraindication to transplant, highlighting the importance of this imaging finding [50].

**FIGURE 7:**
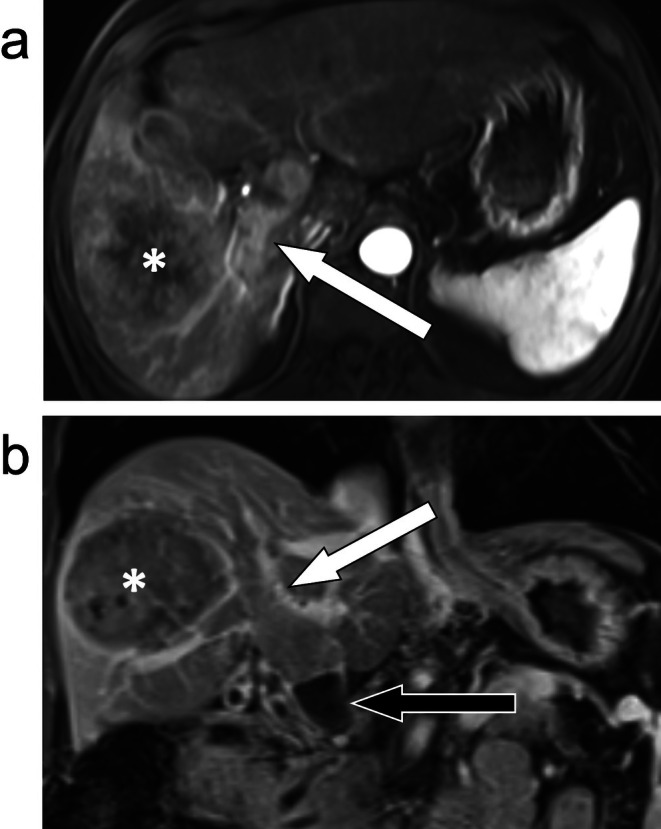
T_1_‐weighted fat‐suppressed arterial phase axial image (a) and portal venous phase coronal image (b) demonstrate definitive enhancement of soft tissue in the right portal vein (white arrows) in contiguity with a malignant parenchymal mass (asterisks). This was categorized as LR‐TIV (tumor in vein). The coronal image also demonstrates an adjacent bland thrombus (black arrow).

Overall performance of this category for identifying tumors with macroscopic venous invasion is good, with 62% sensitivity and 99.8% specificity for MRI in a recent large retrospective study [51]. Unequivocal enhancement of an intravascular filling defect is the most specific feature of tumor in vein and has performed well in retrospective studies [52] (Figure 7). Additional features may be suggestive of tumor in vein but should not be used in isolation, as they may overlap with the appearance of bland thrombus, such as restricted diffusion within a vein [53]. The LI‐RADS manual suggests describing a likely etiology if possible. For example, if the tumor in vein is adjacent to an LR‐5 observation, describing the tumor in vein as “LR‐TIV, probably due to HCC.” Of note, an adjacent mass is not a requirement to assign the LR‐TIV category [1].

## 8 Interobserver Agreement of the Diagnostic Algorithm

Studies evaluating the inter‐reader agreement of LI‐RADS have generally shown promising results for both CEUS [54, 55, 56] and CT/MRI [33, 57, 58]. In a 2019 CEUS study of 1366 patients that involved four readers, Li et al. found moderate to substantial agreement in category assignment (range in *κ*, 0.61–0.73), APHE (range in *κ*, 0.65–0.83), and washout (range in *κ*, 0.58–0.71) [55]. A 2020 systematic review and meta‐analysis evaluated data from eight studies and 1379 observations, finding substantial inter‐reader agreement (summary *κ*, 0.76, 95% CI, 0.67–0.83) [54]. A subsequent study evaluating the effect of reader experience on inter‐reader agreement included two residents, two fellows, and two specialists, and found moderate, substantial, or almost perfect agreement in the evaluation of major features (range in *κ*, 0.54–0.94) [56]. Inter‐reader agreement for major features was almost perfect among specialist radiologists (range in *κ*, 0.81–0.94), and the overall inter‐reader agreement in the assignment of categories was moderate to substantial (range in *κ*, 0.60–0.80) [56].

Similar rates of inter‐reader agreement have been obtained for CT/MRI LI‐RADS categorization and evaluation of major features. In an international, multicenter study, Fowler et al. evaluated the inter‐reader agreement of CT/MRI LI‐RADS v2014 amongst 113 readers and 380 image sets [33]. The intraclass correlation coefficient (ICC) of LI‐RADS category assignment was substantial at 0.67 (95% CI, 0.61–0.71) for CT and 0.73 (95% CI, 0.68–0.77) for MRI. Overall ICCs for major features were almost perfect, as follows: APHE, 0.87 (95% CI, 0.84–0.90); washout, 0.85 (95% CI, 0.81–0.88); and enhancing capsule, 0.84 (0.80–0.87). Using linear mixed models, the authors found that ICCs for LI‐RADS category assignment were borderline higher for community radiologists than for academic radiologists (ICC difference, 0.009, *p* = 0.05), but otherwise, ICCs were not affected by liver expertise, LI‐RADS familiarity, or years of post‐residency experience.

A 2020 systematic review and meta‐analysis on the inter‐reader agreement of LI‐RADS major features on MRI found similar results [57]. Based on data from 15 studies and 2968 observations, Kang et al. found substantial inter‐reader agreement with pooled *κ* values as follows: APHE, 0.72 (95% CI, 0.62–0.82); nonperipheral washout, 0.69 (95% CI, 0.60–0.78); and enhancing capsule, 0.66 (95% CI, 0.58–0.74). The pooled *κ* value for LI‐RADS categorization was 0.70 (95% CI, 0.56–0.85), which is similar to the pooled ICC of 0.73 obtained by Fowler et al. [33]. In another multicenter, international study evaluating inter‐reader reliability of scrollable CT/MRI examinations, Hong et al. also found moderate agreement, with ICC for dichotomized LI‐RADS categories of 0.68 (95% CI, 0.61–0.73) and ICCs for major features ranging from 0.50 to 0.65 [58]. However, the inter‐reader reliability could only be assessed for four AFs and ranged from poor (0.16, 95% CI, 0.03–0.30) for transitional phase hypointensity to moderate (0.58, 95% CI, 0.50–0.66) for mild–moderate T_2_ hyperintensity. Other studies have also shown poor to moderate inter‐reader agreement of AFs [35, 36].

## 9 LR Treatment Response Algorithm

The LI‐RADS TRA provides guidelines for assessing HCC treated with locoregional therapy [59]. The TRA was updated in 2024 and divided into two components, the Radiation TRA and Nonradiation TRA, reflecting distinctly different expected evolution pathways for treatment changes [60, 61, 62]. Radiation‐based modalities (primarily trans‐arterial radioembolization [TARE] and external beam radiation modalities) treat tumors by two different pathways [63]. First, there is immediate cell death due to radiation damage. Second, surviving tumor cells sustain DNA damage such that the cells remain alive and perfused but unable to replicate [64, 65]. For nonradiation‐based treatments, the treatment effect is mainly via immediate cell death [63].

**FIGURE 8:**
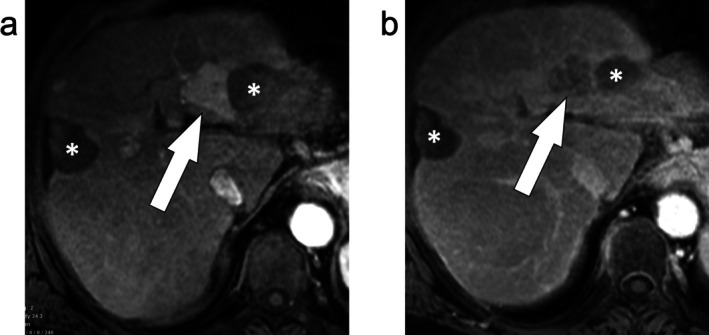
Axial T_1_‐weighted fat‐suppressed post‐contrast arterial phase (a) and portal venous phase (b) images demonstrate two percutaneous ablative zones in the liver left lateral section and segment 8 (asterisks). The ablative zone in the liver left lateral section demonstrates a large masslike area of arterial phase hyperenhancement along the ablative margin (a, arrow) with nonperipheral washout (b, arrow) consistent with viable tumor (LR‐TR Viable). The ablative zone in segment 8 (asterisk) demonstrates a treatment‐specific expected enhancement pattern and was considered nonviable (LR‐TR Nonviable).

The primary imaging manifestation of viable tumor is sustained perfusion of a lesion, as demonstrated by mass‐like enhancement after treatment (Figure 8). As a result of the distinct mechanisms between the two groups of treatment modalities, the interpretation of post‐treatment findings also differs [60].

If a treated observation is completely nonenhancing, the tumor is presumed necrotic, and the observation is categorized as LR‐TR Nonviable [62]. In cases where there is persistent mass‐like enhancement, when the treatment was nonradiation based, viable tumor is presumed to be present, the observation is categorized as LR‐TR Viable, and additional treatment should be considered. When persistent mass‐like enhancement is present following a radiation‐based treatment, however, interpretation must also reflect the observation's imaging history. If the observation is stable or decreasing in size, the tumor cells are presumed viable but unable to replicate. The observation is then categorized as LR‐TR Nonprogressing, and surveillance rather than further treatment is indicated, even though at that snapshot in time the tumor remains perfused.

The Nonradiation TRA also allows for scenarios in which the enhancement of a treated observation is uncertain, in which case the observation is categorized as LR‐TR Equivocal [62]. Several primary papers and a meta‐analysis have shown that more than half of LR‐TR Equivocal observations contain viable tumors and are likely to progress in the future, so further treatment should be considered for these observations [66]. The Radiation TRA does not include a distinct category for uncertain enhancement, and in scenarios where enhancement is uncertain after a radiation‐based treatment, categorization is at the reader's discretion [62]. These observations are often LR‐TR Nonprogressing since they are not definitely nonviable but cannot be confirmed to have increasing mass‐like enhancement.

Finally, both TRAs allow for the optional use of the AFs, including restricted diffusion (any degree) or mild–moderate T_2_ hyperintensity, to upgrade the observation category from LR‐TR Nonprogressing to LR‐TR Viable (Radiation TRA) or LR‐TR Equivocal to LR‐TR Viable (Nonradiation TRA).

## 10 Artificial Intelligence and LI‐RADS

AI models have been applied in radiology for more precise tumor segmentation and automated diagnosis [67]. AI systems could be adapted in LI‐RADS to help standardize categorization, improving consistency across institutions and readers. AI models can go beyond diagnostic tasks, aiding in prognostic assessments and guiding clinical decisions based on more sophisticated analyses [68]. Models could be integrated into LI‐RADS to help inform treatment strategies, making the system a diagnostic tool and a guide for therapeutic decisions, particularly for liver cancer treatments like transarterial chemoembolization (TACE) or surgical interventions [69].

Radiomics and AI have been applied in multiple studies specific to HCC CT and MR imaging [70, 71, 72, 73]. These mostly focus on specific tasks, and it remains extremely challenging to build a fully autonomous algorithm capable of independently analyzing MRI DICOM‐format images to generate a LI‐RADS‐based report similar to that created by a radiologist. There are several challenges.

### 10.1 Large Datasets

It is difficult to build large, well‐labeled datasets of modalities such as MRI in patients at high risk for HCC. This requires many hours of expert review, ideally performed in an independent fashion by multiple experts, with a disagreement resolution mechanism in place to establish ground truth. Interpretation of MRI for HCC using LI‐RADS requires knowledge of anatomy, pathophysiology including normal and abnormal enhancement patterns within the liver, and an understanding of how to apply all five major and 21 ancillary LI‐RADS CT/MRI diagnostic algorithm features. This can be time‐consuming to systematically document. Furthermore, many LR‐5 liver observations do not undergo tissue sampling, which may require an alternative reference standard that can involve medical record chart review and review of follow‐up imaging, further adding to the time required to build a database with an adequate ground truth [74].

### 10.2 MR Image Spatial Localization

**FIGURE 9:**
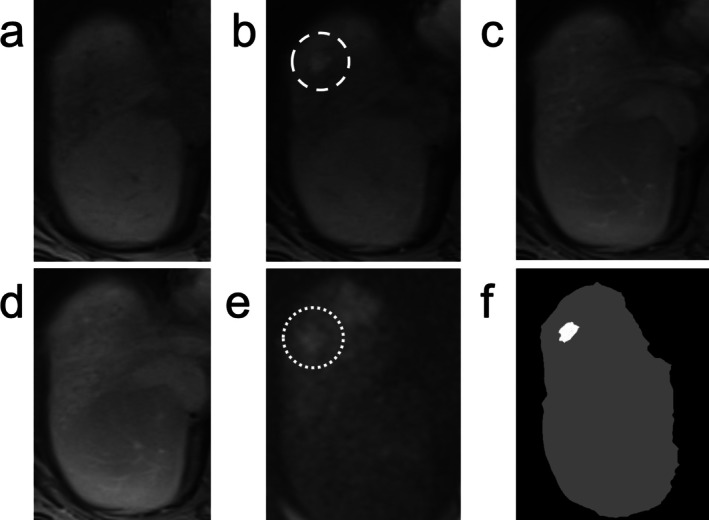
Axial T_1_‐weighted fat‐suppressed imaging (a) does not demonstrate a lesion. Arterial phase imaging (b) demonstrates an observation in hepatic segment 8 (dashed circle), that is not visible on the portal venous phase (c) or 5‐minuted delayed phase (d). This observation was demonstrated on diffusion weighted imaging to be slightly hyperintense (e, dotted circle) and was considered LR‐4. A segmentation map (f) was obtained that demonstrates a white region corresponding to the segment 8 liver observation, and a gray region corresponding to the liver.

An automated algorithm that applies LI‐RADS would need to identify the liver and observations within the liver. While the liver and liver observations can be segmented on a single image, expanding this to all images on a single MRI sequence and then co‐registering segmentation maps across sequences can be particularly challenging due to misalignment between sequences from patient movement, variable breath hold volumes, and other factors that can lead to distortions of the liver, including from rotation and translation. Many liver observations may only be visible on a few sequences, which further complicates aligning focal abnormalities across sequences (Figure 9).

### 10.3 Computational Power

**FIGURE 10:**
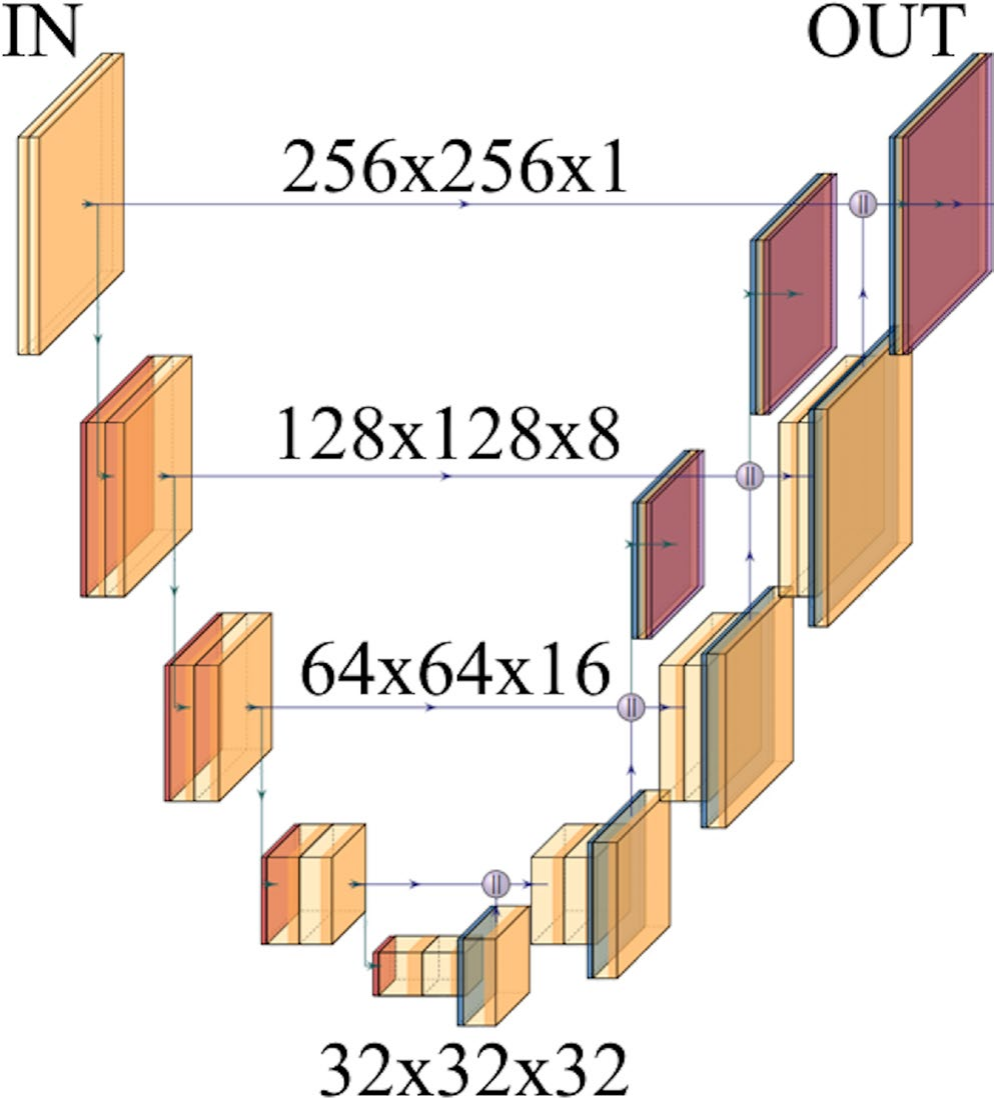
Illustration demonstrating the steps of a typical U‐net convolutional neural network architecture developed for image segmentation. The U‐Net architecture downsamples the input data, reducing the size but increasing the number of AI‐derived features, then upsamples the data back to the original size. This often places constraints on the input image dimensions; in this instance, the network required 2D input with a height and width of 256 pixels.

A data processing challenge exists due to the large size of many medical imaging exams, which often results in the need for machine learning models such as convolutional neural networks to resample images due to computational limits. This is typically done by either assessing smaller segments of images using patch‐based sampling or by downsampling image resolution; for example, some implementations of the U‐Net segmentation algorithm require that DICOM volumes are first reduced to 2D images smaller than 300 × 300 pixels (Figure 10) [75, 76]. Resampling images risks removing data that is important for model performance. Limited data are available regarding the best technique for liver observation sampling and segmentation when applying LI‐RADS.

Given the complexity of preparing large amounts of MR images for machine learning, many models are trained on relatively fewer images. This can increase the risk of overfitting models to the training data [77], which can manifest as models that perform well on their training data but generalize poorly to new datasets. Transfer learning, which can help address data scarcity whereby knowledge learned from one task is reused on a separate but similar task, may improve MRI classification of LI‐RADS on external datasets [78]. Other techniques exist to address small dataset size, such as data augmentation, but each has limitations [79].

Several more recent works have had some success applying AI to LI‐RADS. Mulé et al. found that a machine could classify a liver observation as LR‐5 with high specificity using CT images and could improve a radiologist's visual analysis [67]. Laino et al. conducted a systematic review to determine the added value of AI to LI‐RADS that included 11 studies published up until 2021 and found that machine learning could perform similarly to radiologists at identifying MRI features of liver observations important to LI‐RADS [68]. AI has been used for other aspects of liver imaging not specific to LI‐RADS, such as lesion detection, segmentation, and classification on CT, including in a study on 12,610 patients that classified lesions into one of seven diagnoses (HCC, intrahepatic cholangiocarcinoma, metastasis, hemangioma, cyst, focal nodular hyperplasia, hepatic abscess) and found that AI could improve the performance of radiologists at all stages of their career [80].

In a recent study by Fervers et al., ChatGPT (GPT‐3.5), a large language model, was used to assign LI‐RADS scores based on free‐text and structured radiology reports describing liver observations [81]. While their results were disappointing, with ChatGPT achieving low accuracy for LI‐RADS classification, it is likely that future large language models will continuously improve their ability to understand text in radiology reports and correctly apply systems such as LI‐RADS using radiologist descriptors, which could conceivably reduce misclassification errors by radiologists. In another study, a domain‐specific chatbot was tested on free‐text MRI reports using both real and synthetic liver observations and found that the chatbot could achieve moderate agreement with the ground truth [82]. Future research on how to further optimize large language models for this purpose would be beneficial.

## 11 Points of Controversy

While LI‐RADS has demonstrated numerous strengths and advances in reporting, there remain areas for future improvement. Since its inception in 2011, there has been a large impact on HCC research, but LI‐RADS has not been universally adopted or uniformly applied, with ongoing language translations in an attempt to facilitate broader uptake. A barrier to its uptake may include its perceived complexity. For example, the use of hepatobiliary contrast agents requires a more stringent interpretation of washout, applied only in the portal venous phase, which could lead to decreased use in centers using these agents [1]. Further, in areas with high rates of HCC, such as Asia, the preference for hepatobiliary contrast lies within the desire for high sensitivity, whereas LR‐5 categorization is aimed at preserving extremely high specificity and PPV. As discussed, the larger number of AFs and the nuance of which apply to which modality, which contrast agent, and rules of increasing an observation's categorization are a cause for inter‐reader variability [3]. Ultimately, simpler versions may be of utility in improving generalizability to overcome these barriers. Modified categorizations have been proposed via a simplified version of LI‐RADS, which demonstrated preserved PPVs and specificity but improved sensitivity for LR‐5 when tested across multinational pooled data [42, 83].

Beyond the cognitive interpretive burden of the system, examination of LI‐RADS performance and recommendations raises interesting points. The use of LI‐RADS over the years has resulted in a large accumulation of data; however, changes over its iterations limit the ability to pool such data. Conversion schemas have been proposed to assist in translating research across the different versions to overcome this limitation [84]. When examining the available existing data, it is clear LI‐RADS performs very well, particularly for categories 1, 4, 5, and M [25, 85, 86]. However, the diagnostic performance of the LR‐2 and LR‐3 categories is less clear. Pooled observed percentages of HCC in LR‐2 and LR‐3 are higher than expected, ranging from 1% to 14% for LR‐2 and 31%–38% for LR‐3 [85, 86, 87]; this raises a question of selection and verification biases, with possible increased reporting of LR‐2 solid nodules rather than presumed vascular shunts and increased biopsy rates of the same [74, 85]. Thus, a more accurate assessment of the performance of the LR‐2 category is needed. Given that the rates of HCC in LR‐3 observations are also high at approximately 38% [85, 87], and that biopsies are performed elsewhere in the body for similar or even lower rates of malignancy, these data raise concerns as to whether current management guidelines for LR‐2 and LR‐3 observations, namely return to surveillance and imaging follow‐up, are appropriate. Biopsy may have an expanded role across LI‐RADS categorizations even in LR‐5 lesions to assist in the stratification of subtype and histologic features due to the impact on treatment decisions [10].

Ultimately, LI‐RADS has positively impacted the landscape of HCC reporting and patient care. Its continued improvement over time will depend on ongoing quality clinical, histologic, and radiologic evidence.

## 12 LI‐RADS and Other Non‐Invasive HCC Diagnostic Systems

LI‐RADS has been increasingly adopted by a variety of organizations and incorporated into their recommendations, including the AASLD, OPTN, and the European Association for the Study of the Liver (EASL). Several other associations continue to use their own systems, including the Asian Pacific Association for the Study of the Liver (APASL), the Japan Society of Hepatology (JSH), and the Korean Liver Cancer Association‐National Cancer Center (KLCA‐NCC) [88, 89, 90]. Unlike LI‐RADS, the APASL, JSH, and KLCA‐NCC systems permit the classification of washout in the transitional and hepatobiliary phases, in addition to the portal venous phase, when using hepatobiliary contrast agents. Studies have found that this may improve sensitivity for “definite HCC,” with a possible drop in specificity; however, more research is needed [91, 92].

## 13 Future LI‐RADS Iterations

Since 2011, ACR LI‐RADS [1] has seen several updates to improve diagnostic accuracy for HCC [93]. Despite its widespread use, areas for improvement remain, particularly concerning the role of AFs [94].

Recent studies highlight the complexity surrounding the role of AFs in the LI‐RADS system. A recent IPD meta‐analysis showed that most AFs are independently associated with their intended use; however, their overall impact on LI‐RADS as a whole is less clear [37]. Future iterations of LI‐RADS may focus on simplifying the diagnostic algorithm by refining or limiting the use of AFs, which currently add inter‐reader and inter‐modality variability without clear diagnostic benefit.

A critical area for future iterations of LI‐RADS is addressing variability in how major features are combined to categorize liver observations. A systematic review highlighted substantial heterogeneity in diagnostic accuracy, with I‐squared values for the LR‐3, LR‐4, and LR‐5 category PPVs ranging from 33% to 67%, indicating moderate to high levels of heterogeneity [85]. This suggests variability of the PPV of major feature combinations within each LI‐RADS category. The LI‐RADS IPD group is currently evaluating the PPV of different major feature combinations to determine whether some warrant recategorization [42, 83, 95]. This ongoing work is crucial, as improved categorization could reduce variability, enhance diagnostic accuracy, and lead to better outcomes for patients at risk for HCC.

An ongoing data collection strategy includes work by the LI‐RADS IPD Group, who continuously seek to collect more primary study data on LI‐RADS, which can be used to then conduct IPD meta‐analyses. Future planned areas of research include exploring the impact of treating transitional phase and hepatobiliary phase hypointensity the same as nonperipheral washout in the LI‐RADS algorithm, identifying noncontributory ancillary features using machine learning, and clarifying global reporting patterns of LI‐RADS research. Simplifying LI‐RADS may lead to improved inter‐observer agreement and even broader uptake. The prospective collection of data, and ultimately images, into a central registry would be helpful to continuously evaluate LI‐RADS.

Machine learning models offer promising avenues to enhance LI‐RADS. For example, machine‐driven decision tree models can automate liver observation segmentation and classification, reducing inter‐reader variability and improving diagnostic performance [96, 97]. Studies have shown that machine learning models can help identify noncontributory AFs and focus diagnostic algorithms on the most predictive variables, streamlining complexity and increasing efficiency [43]. Beyond classification and segmentation, AI holds the potential to support broader oncological applications, as demonstrated in research on AI‐driven decision making in oncology [71]. The integration of machine learning, radiomics, and other biomarkers such as serum alpha‐fetoprotein into LI‐RADS may help improve performance, but at present, LI‐RADS is based entirely on radiologist interpretation of images.

## 14 Conclusion

LI‐RADS is the preeminent system for classification and risk stratification of liver observations on imaging in patients at high risk for HCC. LI‐RADS is derived from a combination of evidence and expert opinion. As the evidence body grows, LI‐RADS continues to evolve to improve diagnostic accuracy and reproducibility, with the goal of ultimately improving patient outcomes.
